# Determination of chloramphenicol in meat samples using liquid chromatography–tandem mass spectrometry

**DOI:** 10.1002/fsn3.2530

**Published:** 2021-08-16

**Authors:** Salma Akter Mou, Rafiza Islam, Mohammad Shoeb, Nilufar Nahar

**Affiliations:** ^1^ Department of Chemistry University of Dhaka Dhaka Bangladesh

**Keywords:** antibiotic residues, beef samples, chloramphenicol, liquid chromatography‐tandem mass spectrometry, poultry meat samples

## Abstract

Chloramphenicol (CAP), a bacteriostatic antibiotic, is used for the treatment of bacterial infections in human and animals. Continual exposure of CAP residues into animal tissues may lead to antibiotic resistance. For the protection of humans and animals from this problem, a fast and highly sensitive analytical method based on ultra‐high‐performance liquid chromatography–tandem mass spectrometry (UHPLC–MS/MS) was developed and validated in this study for the quantitative determination of CAP in poultry meat and beef samples. Quick, easy, cheap, effective, rugged, and safe (QuEChERS) method was used for the extraction of CAP residues. The developed method was validated in terms of linearity, accuracy, precision, and specificity. Poultry meat and beef samples were extracted with 20 ml water–acetonitrile (1:1, v/v) and cleaned up by MgSO_4_, primary secondary amine, and C18 powder. The method was found to be linear in a wide concentration range, with correlation coefficient of higher than 0.999. The repeatability and reproducibility of this method were satisfactory. The achieved limit of detection and limit of quantification were 0.16 and 0.50 ng/g, respectively. Recoveries were estimated at 5 and 10 ng/g spiking levels in the range of 99%–111% with the coefficient of variation 0.48%–12.48% for spiked samples, and the matrix enhancement effects were mild in the range of 80%–85%. In this study, the levels of CAP residue in tested real samples were found below the detection limit. The method proved to be suitable for CAP determination in all kinds of samples tested and also efficient for the application of routine analysis.

## Graphical abstract

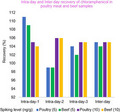

## 1 INTRODUCTION

**FIGURE 1:**
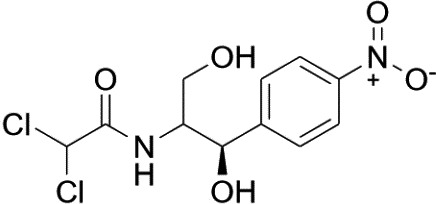
Chemical structure of chloramphenicol (CAP)

Chloramphenicol (CAP; C_11_H_12_Cl_2_N_2_O_5_; Figure 1) is a broad‐spectrum antibiotic having activity against both gram‐positive and gram‐negative bacteria and is effective in the treatment of several infectious diseases in animals all over the world, including food‐producing animals because of low cost, great pharmacokinetics properties (Zhiming et al., 2015), remarkable penetration into the tissues, and ready availability (Rønning et al., 2006). However, CAP is, in certain susceptible individuals, associated with harmful effects in human such as bone marrow depression, fatal aplastic anemia, leukemia, allergic reactions, and gastrointestinal disorder (Mbodia et al., 2014; Mehdizadeh et al., 2010; Rønning et al., 2006). As a consequence, CAP has been banned for use in food‐producing animals in EU, China, United States (Imran et al., 2017). Later, CAP is included in Annex IV of Council Decision 2077/90 (Council Regulation (EEC) 1990), which comprises the antibiotic with an established zero‐tolerance level in edible tissues. Food and Agricultural Organization and World Health Organizations have also announced that the application of CAP is not allowed in poultry meat and beef neither a maximum residue limit considering the existence of fatal dose‐independent effects (Bakar et al., 2013; Nicolich et al., 2006; Raffi and Suresh, 2011). But unfortunately, it is easily available in Asia and broadly used for livestock and aquaculture (Bakar et al., 2013). In order to monitor and strict control, the residual level of CAP, sensitive and accurate analytical methods are needed. Capillary electrophoresis, gas chromatography–mass spectrometry (GC‐MS), gas chromatography with electron capture detection (GC‐ECD), radio immune assay and enzyme immunoassay, microbiological methods, liquid chromatography‐tandem mass spectrometry (LC‐MS/MS) etc. were used for the screening, confirmatory, and other analytical methods for determining CAP in earlier days (Rønning et al., 2006). Capillary electrophoresis method is not suitable for routine analysis due to low precision (Blais et al., 1994). Microbiological methods involving bacteriological growth inhibitor tests required several days for analysis and not enough sensitive (Rajia et al., 2020). Methods involved GC‐MS, GC‐ECD required derivatization of CAP to decrease its polarity which is time consuming and may affect recovery experiments (Li et al., 2006). Immunoassay method is advanced method for analysis of CAP, but this method is not suitable due to the possibility of obtaining false‐positive results arises from matrix interference (Chuanlai et al., 2005). Liquid chromatography‐tandem mass spectrometry methods have been widely used nowadays for analysis of antibiotic residues due to high sensitivity and selectivity. So, the aim of this study was to develop a rapid, reliable, and user‐friendly LC‐MS/MS coupled with electrospray ionization (ESI) system and triple quadrupole (QQQ) mass analyzer‐based screening and confirmatory method for identification and quantification of residual CAP in poultry meat and beef samples collected from local markets and super shops of Dhaka city with good selectivity, high sensitivity, fine precision, and accuracy and validate the method along the guidelines given in 2002/657/EC to control the food safety for human consumption.

## 2 MATERIALS AND METHODS

### 2.1 Chemicals and reagents

Certified standard, that is, CAP, reversed‐phase silica gel (C18, particle size 5 µm), LC‐MS/MS grade methanol (MeOH), and acetonitrile (ACN) were purchased from Sigma‐Aldrich, USA. Primary secondary amine (PSA) was purchased from Supelco, USA.

### 2.2 Liquid chromatography–mass Spectrometer instrumentation

Analysis was performed using liquid chromatography–mass Spectrometer (LC‐MS/MS, Model Shimadzu‐8050) coupled with ESI system and QQQ mass analyzer. Ultra‐fast liquid chromatography (LC; Model‐ Shimadzu Prominence) system contained column oven (Model‐ CTO‐10AC), auto sampler (Model‐ SIL‐20AC HT), and shim‐pack GISS C18 reversed‐phase column (250 × 4.6 mm i.d.; particle size 5 µm). N_2_ gas (drying & nebulizing gas), air (heating gas), and Ar gas (collision gas) were used for sample analysis.

### 2.3 Liquid chromatography conditions

Chromatographic separation of CAP was achieved using C18 reversed‐phase column operating at column oven temperature of 40℃. MeOH:H_2_O with 0.1% formic acid (30:70 ratio) was used as a mobile phase operated in an isocratic elution condition. The flow rate of the mobile phase was 1.0 ml/min, and the injection volume was 20 µl for standard and samples.

### 2.4 MS/MS conditions

The mass spectrometry analysis mode was negative scan for identification with the following conditions: flow rate of nebulizing gas, drying gas, heating gas was 3, 10, and 10 L/min, respectively, and temperature of interface, de‐solvation line, and heat block was 300℃, 250℃, and 400℃, respectively. The negative multiple reaction monitoring (MRM) mode was used for quantification using *m*/*z* 321 → 152 (product ion) for CAP at retention time 7.09 min.

### 2.5 Sample collection

Poultry meat (*n* = 30) and beef samples (*n* = 30) were collected from five different markets and five super shops of in Dhaka city. Each sample (10 g) was homogenized using kitchen blender and was taken in screw cap Teflon tube (50 ml) and stored at −20℃ until analysis.

### 2.6 Standard preparation

CAP stock solution of 1000 mg/L was prepared by taking 0.01 g CAP standard in 10 ml volumetric flask followed by making up to the mark with deionized water (H_2_O), and then CAP primary standard solution of 10 mg/L was prepared consequently. Finally, a series of CAP working standard solution with concentrations in the range of 0.05–100 ng/ml was prepared. A mixture of solvents between MeOH and H_2_O at a 30:70 v/v ratio was used.

### 2.7 Extraction

Homogenized samples (10 g) were extracted by using quick, easy, cheap, effective, rugged, and safe (QuEChERS) method with some modification. In brief, the sample was extracted using H_2_O and ACN (20 ml, 1:4 ratio) mixture of LC‐MS/MS grade and vortexed about 5 min for homogeneous mixing. Then, for the separation of phase, mixture of MgSO_4_ (4.00 g) and NaCl (1.00 g) was added to the mixture and again vortexed for 10 min followed by centrifuged at 6000 rpm for 10 min. The CAP containing upper ACN layer was transferred into volumetric flask (20 ml in size) and diluted with ACN to its volume mark. The ACN solution was then transferred into a centrifuge tube (50 ml) with addition of hexane (20 ml), vortexed for 5 min, followed by centrifugation at 6000 rpm for 10 min. The hexane layer was discarded. One milliliter of the ACN solution was transferred into a LC sample vial (2ml), and a mixture of MgSO_4_ (150 mg), PSA (50 mg), and C18 (50 mg) were added, vortexed for 2 min, and filtered through nylon sample filter (0.22 µm) into another sample vial for analysis with LC‐MS/MS.

### 2.8 Analytical method validation

The method was validated by following the EU Commission Decision, 2002/657/EC (European Commission, 2002). Specificity was confirmed by injecting control samples extract and selectivity was evaluated by analyzing standard CAP, blank matrices, and sample matrices spiked with CAP simultaneously and monitoring retention time. Unwanted components interfering with analytes were analyzed by comparing the chromatogram of the standard CAP, blank matrices, and matrices spiked with CAP.

#### 2.8.1 Limit of detection (LOD) and limit of quantification (LOQ)

The lowest concentration of CAP matrix‐matched standard solution which instrument can detect was used for the determination of LOD. Then, this CAP matrix‐matched standard solution was analyzed for 3 times. The standard deviation of the response (peak area) was used for calculation with linear equation obtained from calibration curve. The obtained concentration was then multiplied with 3 for LOD and with 10 for LOQ based on statistical method (Indrayanto, 2018).

#### 2.8.2 Linearity

Linearity was carried out at six concentrations ranging from 0.05 to 20 ng/g. A calibration curve was constructed by plotting the peak area versus concentration.

#### 2.8.3 Accuracy and precision

The accuracy was evaluated in terms of percentage recoveries of each sample which calculated from matrix‐matched calibration curve and matrix effect was calculated by comparing with that of calibration curve of standard CAP with mobile phase and matrix‐matched. For recovery experiment, poultry meat sample (10 g) was taken in Teflon tube. Samples were spiked with CAP standard solutions at 5 and 10 ng/g levels for repeatability (intraday) and reproducibility (interday), and the sample was allowed to stand for 1 hr to let the antibiotic to be absorbed into the samples. The precision of the method was estimated by determining the coefficient variation (CV).

#### 2.8.4 Matrix effect

Matrix effect (%) was calculated with reference to peak area of matrix of control sample and peak area of standard solvent. To evaluate matrix effect, matrix‐matched calibration was used.

## 3 RESULTS AND DISCUSSION

### 3.1 Method development and optimization

**FIGURE 2:**
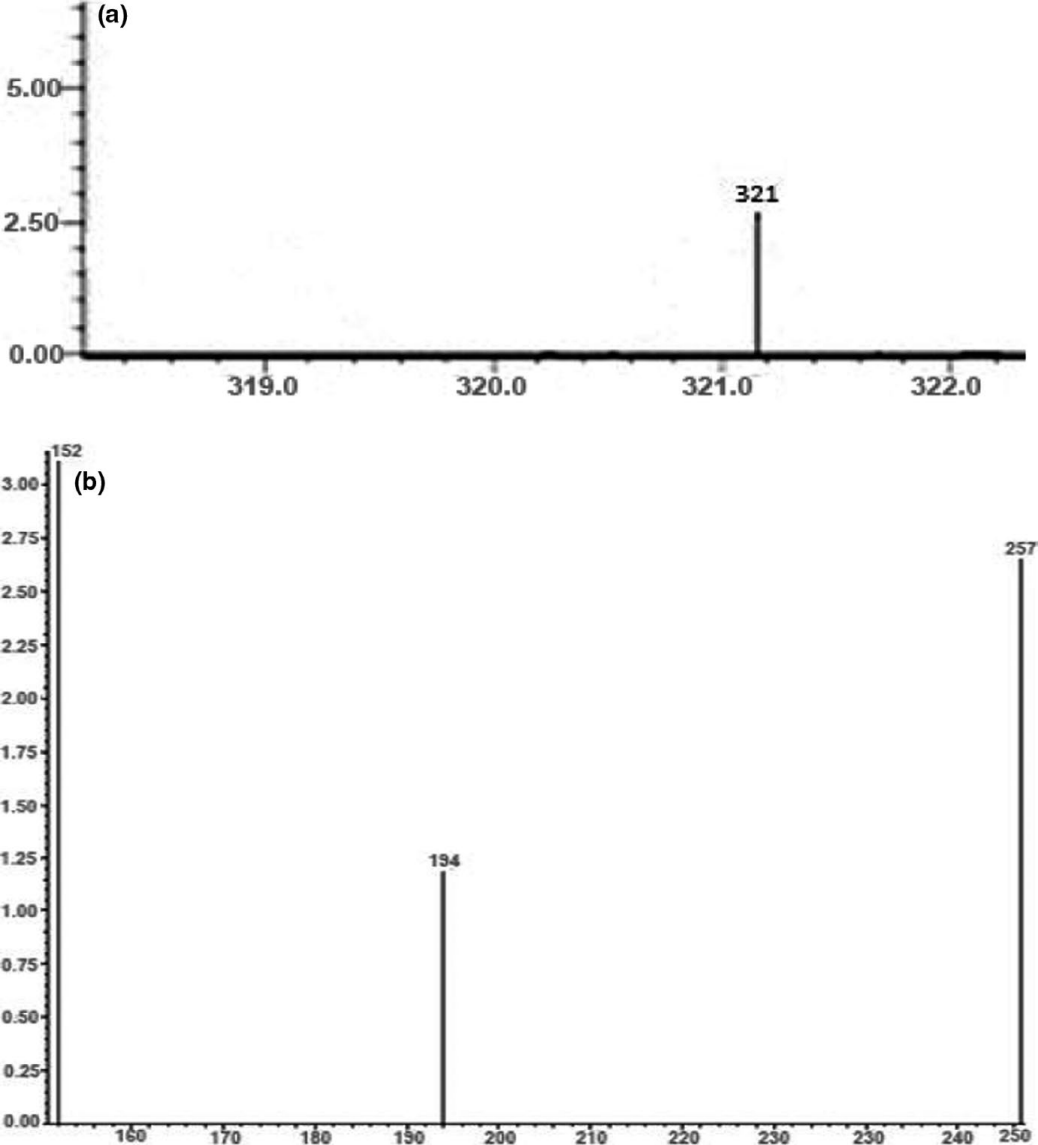
LC‐MS/MS spectrums of (a) CAP precursor ion, (b) product ions

**FIGURE 3:**
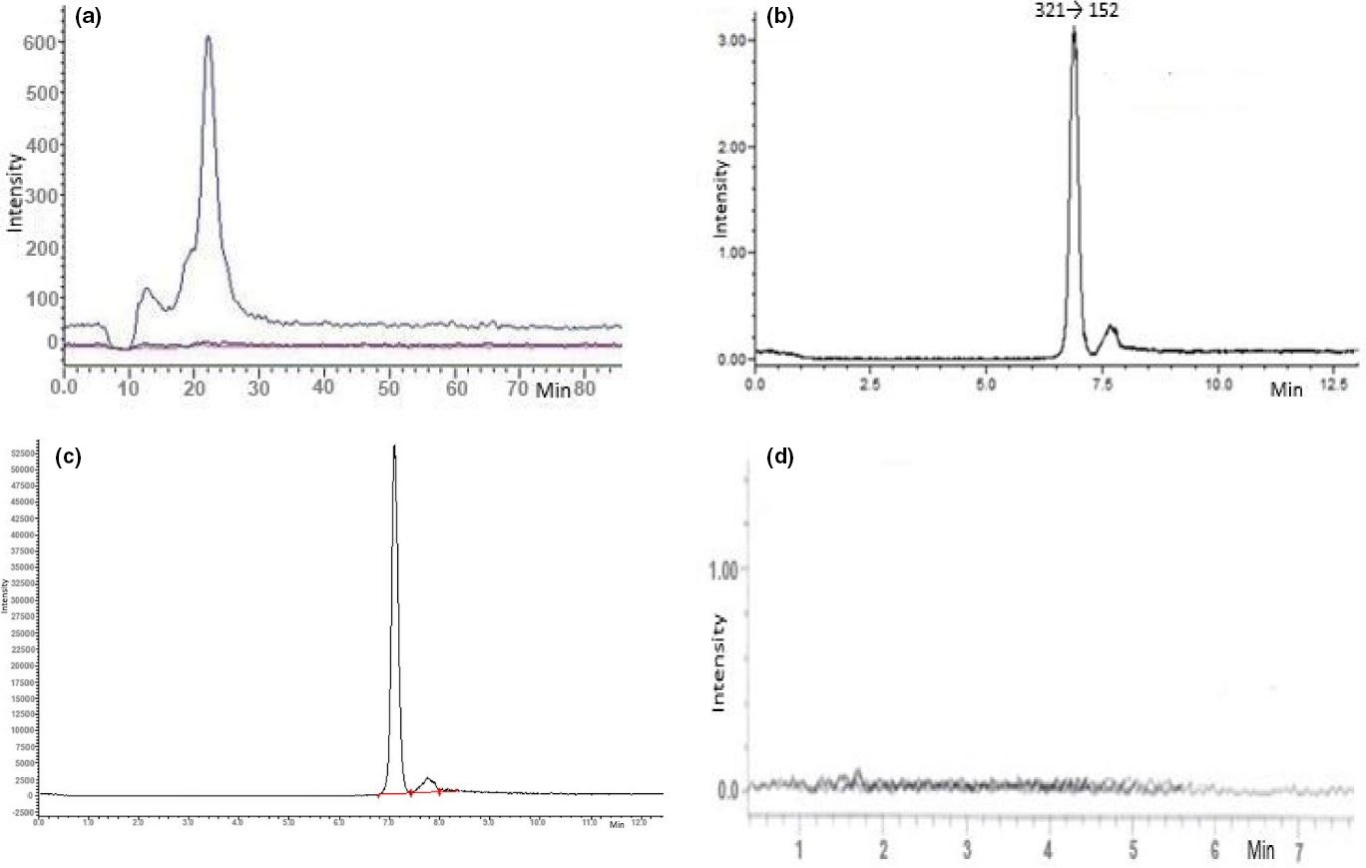
MRM chromatograms of (a) solvent blank, (b) CAP standard, (c) spike sample, (d) poultry meat sample

At first, in this analysis precursor ion (321) was identified using flow injection method in Q3 scan mode without using any collision energy (Figure 2). The parent and product ions were first optimized by injecting a 250 ng/ml standard solution of CAP in both positive and negative polarity mode. Due to deprotonation of CAP, the intensity of precursor ion was much higher in negative mode. After that, optimization of the MS/MS parameters was performed (Table 1). Three characteristic fragmentations of the product ions (152, 194, and 257) were monitored applying collision energy using MRM event optimization method (Figure 2). Later separations were performed by passing sample through LC column where retention time of CAP was 7.09 min (Figure 3b).

**TABLE 1:** Multiple reaction monitoring (MRM) method for product ions and voltage parameters of detection of chloramphenicol

Name	Retention time (min)	MRM method (*m*/*z*)		Voltage (*V*)		
		Precursor ion [M‐H]^‐^	Product ion	Q1 Pre Bias	CE	Q3 Pre Bias
Chloramphenicol	7.09	321	152	17	18	14
			194	17	12	18
			257	17	11	25

### 3.2 Method validation

#### 3.2.1 Linearity

The linearity was checked for standard solutions containing total CAP in the range from 0.5 to 20 ng/g. The square of the correlation coefficient (R^2^) for CAP was 0.999 and 0.999 for matrix‐matched and standard calibration curve, respectively. LOD and LOQ values were 0.16 and 0.50 ng/g, respectively.

#### 3.2.2 Accuracy and precision

The recoveries of the CAP ranged between 99% and 111%. The precision of the method was estimated by determining the CV calculated from results generated under the intra‐(*n* = 5) and interday (*n* = 15), and CV was found in the range between 0.48% and 12.48% (Table 2).

**TABLE 2:** Intraday and Interday recovery of chloramphenicol in poultry meat and beef samples

Sample	Spiking level	Intraday‐1 (*n* = 5)		Intraday‐2 (*n* = 5)		Intraday‐3 (*n* = 5)		Interday (*n* = 15)	
	(ng/g)	Recovery (%)	CV (%)	Recovery (%)	CV (%)	Recovery (%)	CV (%)	Recovery (%)	CV (%)
Poultry meat	5	111	3.97	99	6.32	104	12.26	105	7.59
	10	105	0.48	106	2.97	105	3.04	105	2.29
Beef	5	109	3.77	99	6.32	102	12.48	104	7.63
	10	104	0.57	106	3.02	105	3.07	105	2.32

#### 3.2.3 Matrix effect

For the CAP, the matrix enhancement effects were mild in the range of 80%–85%. From this result, it was concluded that samples matrix interfered with the detection of CAP. So, matrix‐matched calibration curves were used for quantitative analysis.

### 3.3 Analysis of real samples

The validated LC‐MS/MS method was used to analyze collected thirty poultry meat and thirty beef samples (every three replicates). The levels of CAP in tested samples were found below the detection limit. One of the main reasons might be due to the different digestive systems of cattle and fowl than humans (Browne, 1922). One recent report showed that antibiotic was identified in wastes of food animal rather than their meat. CAP is a polar compound and excreted by food animals, and the drug was found in the animal wastes. Manure of the waste of food animals from the agricultural field was up‐taken by vegetables, radish grown in that soil (Chung et al., 2017). Another explanation might be that the withdrawal period of the antibiotic was properly maintained in the poultry firm.

## 4 CONCLUSION

The method described above is sufficiently sensitive and reproducible in the routine analysis of CAP in poultry meat and beef samples within a short analysis time. This method can be used for the improvement of food safety in Bangladesh and other developing countries.

## CONFLICTS OF INTEREST

The authors declared no potential conflicts of interest with the present study.

## AUTHOR CONTRIBUTIONS

**Salma Akter Mou:** Data curation (lead); Formal analysis (lead); Investigation (lead); Methodology (lead); Software (equal); Validation (lead); Writing‐original draft (equal). **Rafiza Islam:** Data curation (supporting); Investigation (supporting); Methodology (supporting); Software (supporting); Validation (supporting); Writing‐original draft (supporting). **Mohammad Shoeb:** Conceptualization (lead); Funding acquisition (lead); Project administration (lead); Resources (supporting); Supervision (supporting); Validation (equal); Visualization (supporting); Writing‐original draft (supporting); Writing‐review & editing (supporting). **Nilufar Nahar:** Conceptualization (supporting); Funding acquisition (supporting); Project administration (supporting); Resources (supporting); Supervision (lead); Validation (supporting); Visualization (supporting); Writing‐review & editing (supporting).

## ETHICAL APPROVAL

There was no necessity for human or animal testing in this study.
